# A chimeric murine coronavirus with altered deISGylase and proteolytic activities exhibits attenuated viral pathogenesis

**DOI:** 10.1128/jvi.00857-26

**Published:** 2026-09-30

**Authors:** Mudassir S. Ali, Amornrat O'Brien, Enya Qing, Lucky Sarkar, Fernando R. Moreira, Bridget S. Banach, Timothy E. O'Brien, Michaela U. Gack, Susan C. Baker

**Affiliations:** 1 Department of Microbiology and Immunology, Stritch School of Medicine, Loyola University Chicago 12248, Maywood, Illinois, USA; 2 Florida Research and Innovation Center, Cleveland Clinic 2569, Port Saint Lucie, Florida, USA; 3 Department of Pathology, Delnor Hospital-Northwestern Medicine 3493, Geneva, Illinois, USA; 4 Department of Mathematics and Statistics, Loyola University Chicago 542491, Chicago, Illinois, USA; The Ohio State University, Columbus, Ohio, USA

**Keywords:** PLP2, PLpro, deISGylation, ISG15, coronavirus

## Abstract

All coronaviruses encode one or two multifunctional papain-like proteases (PLPs) with protease activity that cleaves the viral polyprotein, along with deubiquitinating (DUB) and deISGylase activities that remove ubiquitin and interferon-stimulated gene 15 (ISG15) from viral and host proteins. Studying the role of coronavirus deISGylase activity in viral infection and pathogenesis has been challenging due to the inability to generate a viable deISGylase-deficient coronavirus. To address this issue, we used a recently characterized PLP domain from human coronavirus HKU1 that has intact protease and DUB activity, but minimal deISGylase activity. Switching the HKU1 PLP2 domain with the mouse hepatitis virus strain A59 (MHV-A59) PLP2 domain allowed us to generate a viable chimeric virus, MHV-HP2, that replicated to high titers in cell culture. Analysis of the PLP activities of MHV-HP2 revealed that the virus was impaired in deISGylase activity, intact in its DUB activity, and delayed in proteolytic processing at early time points. Detailed analysis of MHV-HP2 replication kinetics revealed that the delay in proteolytic processing coincided with a transient replication delay in cell culture. This replication delay was not rescued by knockdown of ISG15, suggesting proteolytic delay rather than protein ISGylation drives the delay in replication. Importantly, we found that MHV-HP2 is attenuated for replication and pathogenesis *in vivo,* with infected mice showing more rapid clearance of MHV-HP2 and reduced evidence of liver damage and inflammation as compared to MHV-A59-infected mice. These results document the generation of a murine coronavirus with diminished deISGylase activity and altered protease activity that highlight the importance of coronavirus PLP in coronavirus pathogenesis.

**IMPORTANCE:**

Coronaviruses are adept at circumventing host antiviral defenses by encoding the multifunctional papain-like protease (PLP). PLP modulates the host cellular environment with its proteolytic, deubiquitinase, and deISGylase activities. Coronavirus deISGylase activity targets the host protein interferon-stimulated gene 15 (ISG15), which can be conjugated to host and viral proteins to promote an antiviral state. To assess the contribution of viral deISGylase activity to pathogenesis, we generated a chimeric mouse coronavirus lacking robust deISGylase activity. Characterization of the chimeric virus revealed that the virus was also inefficient in proteolytic processing. We determined that diminished deISGylase activity and altered PLP protease activity resulted in attenuated pathogenesis in a mouse model system, since mice infected with the chimeric virus exhibited rapid clearance of infection and less severe tissue damage as compared to infection with the parental virus. These results reveal that modulating coronavirus PLP deISGylase and proteolytic activities can reduce disease burden.

## INTRODUCTION

Coronaviruses, including the recently emerged severe acute respiratory syndrome coronavirus-2 (SARS-CoV-2) responsible for the COVID-19 pandemic, have evolved multiple mechanisms to subvert and delay the host response to viral infection (1–4). One such mechanism is mediated by a multifunctional papain-like protease (PLP) encoded within the coronavirus replicase polyprotein (5–8). All coronaviruses encode either one or two PLPs within their genome. SARS-CoV-2, SARS-CoV, and Middle East respiratory syndrome coronavirus express one PLP domain, designated PLpro, which is analogous to the PLP2 domain of other coronaviruses (9, 10). Coronavirus PLP domains are multifunctional with catalytic residues mediating the ability to process the viral replicase polyprotein and remove ubiquitin or interferon-stimulated gene 15 (ISG15), a ubiquitin-like molecule, from viral and host substrates (11, 12). Biochemical studies using purified coronavirus PLpro/PLPs have documented the multifunctionality of these enzymes (6–9, 13–18). However, it has been challenging to evaluate the relative contribution of the different enzymatic activities, particularly the deISGylase function, to coronavirus replication and pathogenesis due to the inability to successfully recover a deISGylase-deficient coronavirus. This likely reflects the challenge of modulating coronavirus PLP activity such that only its deISGylase activity is altered without interfering with its ability to process its own polypeptide (17, 19, 20).

Further understanding the role of coronavirus deISGylase activity is important because it has been shown that one target of PLP-mediated deISGylation is the cytoplasmic innate immune sensor melanoma differentiation-associated gene 5 (MDA5) (21). MDA5 is ISGylated at two lysine residues (K23 and K43), and ISGylation of MDA5 is critical for initiating the interferon (IFN) induction pathway (21, 22). Two recent studies documented that coronavirus-mediated deISGylation of MDA5 delays the ability of MDA5 to initiate a type I IFN response in the face of viral infection (21, 22). These findings add to the growing body of literature highlighting the key role of ISGylation in mitigating viral replication (23–27).

A breakthrough in our understanding of the multifunctional nature of coronavirus PLPs came with a recent study that profiled the functional diversity of coronavirus PLPs (28). The authors biochemically purified PLPs expressed by different coronaviruses and evaluated their protease, deubiquitinase (DUB), and deISGylase activities. They revealed a significant variation among the coronavirus PLP domains in their affinity for ISG15 and consequently their deISGylase activity. Of note, the PLP domain with the lowest affinity for ISG15 and with minimal deISGylase activity was from the human coronavirus HKU1 (28). The human coronavirus HKU1 was identified in 2004 as an endemic coronavirus that was associated with respiratory disease primarily in elderly patients and children (29, 30). We hypothesized that deISGylase activity contributes to coronavirus pathogenesis. To test this hypothesis, we sought to recover and evaluate a deISGylase-deficient murine coronavirus by replacing the PLP2 domain of mouse hepatitis virus strain A59 (MHV-A59) with the deISGylase-deficient PLP2 of HKU1, generating a chimeric virus, named MHV-HP2.

Here, we report that chimeric virus MHV-HP2 replicates to high titers in immortalized cell lines, like the parental virus MHV-A59. Analysis of MHV-HP2 PLP activity revealed that it is impaired in its deISGylase activity and retains its DUB activity, but it is delayed in its protease activity. Detailed kinetic analysis revealed that the delay in proteolytic processing corresponded to a transient delay in replication in cell culture. We compared viral replication kinetics, cytokine responses, and liver necrosis in mice infected with MHV-A59 and MHV-HP2 and found that the chimeric virus is significantly attenuated compared to MHV-A59 *in vivo*. This work demonstrates that ablated deISGylase and altered protease activity contribute to attenuation of coronavirus replication and pathogenesis.

## RESULTS

### Evaluating coronavirus PLP deISGylase activity in cell culture

**Fig 1:**
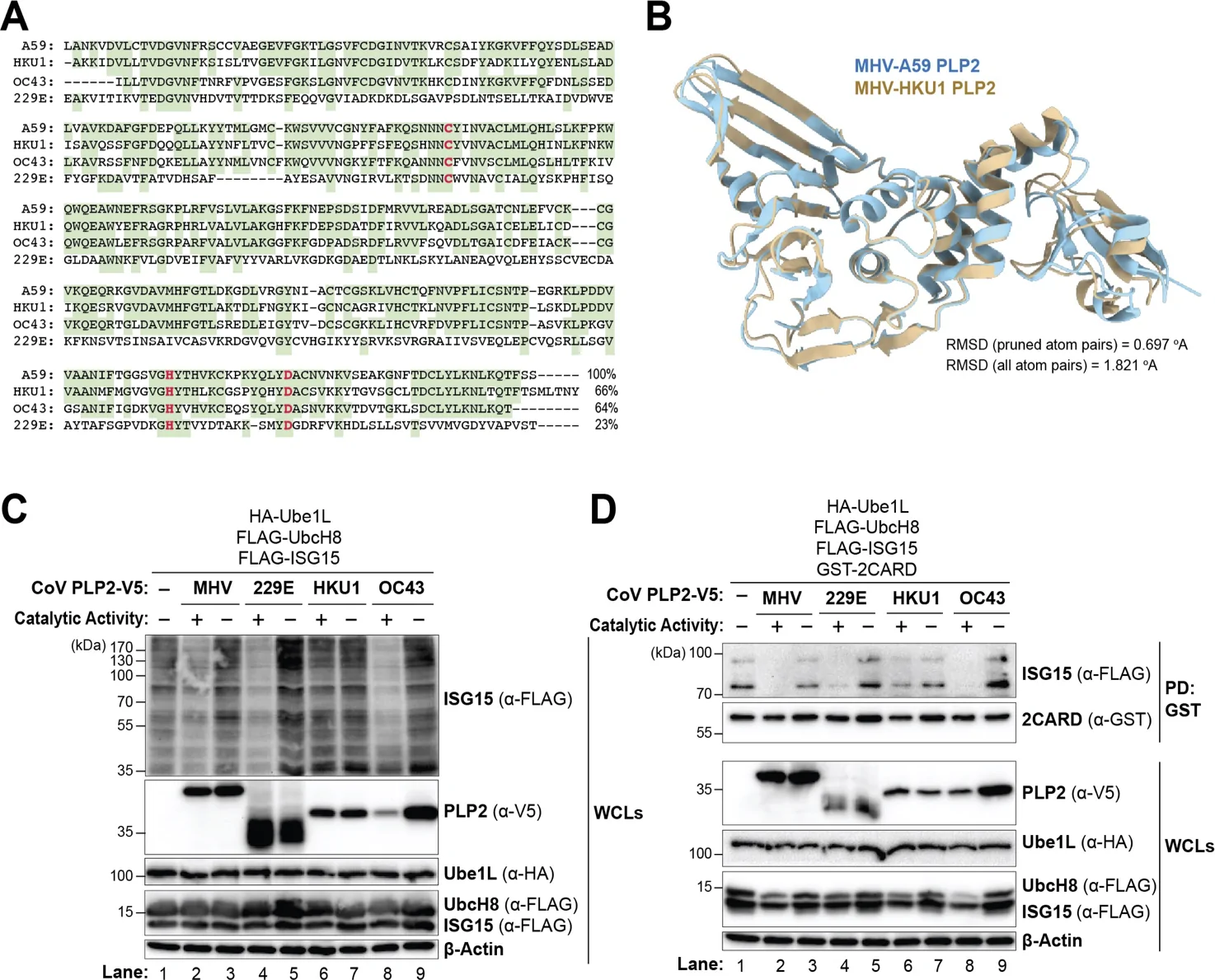
HKU1 PLP2 has a high degree of structural homology to MHV-A59 PLP2 while possessing low deISGylase activity. (**A**) The amino acid sequences of the PLP2 regions of the indicated coronaviruses were aligned, and their percent identity to MHV-A59 was calculated. The catalytic active-site residues are highlighted in red. (**B**) The structures of MHV-A59 PLP2 (PDB 5WFI) and HKU1 PLP2 (PDB 7WFC) were aligned using the ChimeraX Matchmaker function. (**C**) HEK293T cells were transiently transfected with DNA plasmids encoding components of the ISGylation machinery (HA-Ube1L, FLAG-UbcH8, and FLAG-ISG15) along with the indicated coronavirus proteases or empty vector (−) for 24 hours. Whole-cell lysates (WCLs) were assayed via Western blot for the indicated proteins. (**D**) HEK293T cells were transiently transfected with DNA plasmids encoding components of the ISGylation machinery and GST-MDA5 2CARD (GST-2CARD), along with the indicated coronavirus proteases for 24 hours. WCLs were subjected to GST pulldown (PD) and then assayed via Western blot for the indicated proteins. These Western blot results (C and D) are representative of three independent experiments.

Coronavirus PLPs are multifunctional, and isolating the specific role of deISGylase activity has been challenging given the involvement of the same catalytic residues in all three PLP functions. Thus, it has been challenging to generate a viable virus with attenuated deISGylase activity. Here, we capitalized on a previous report of natural variation in the deISGylase activity among six different human coronavirus papain-like proteases (28). This study reported that human coronavirus HKU1 PLP2 had the lowest affinity for ISG15 and the lowest ability to subvert the host IFN response. Important for our work, HKU1 PLP2 has the highest sequence similarity to our model BSL-2 coronavirus, MHV-A59, among the PLP2 proteins we analyzed (Fig. 1A). Additionally, we noted remarkable structural homology between the PLP2s of HKU1 and MHV-A59, with a root-mean-square deviation of 1.821 Å between the two enzymes (Fig. 1B).

We wanted to determine if HKU1 PLP2 was attenuated in its deISGylase activity compared to MHV-A59 in the cellular environment, as this direct comparison was not previously made. To compare deISGylase activities, we transiently transfected HA-tagged Ube1L (E1) and FLAG-tagged UbcH8 (E2) enzymes in HEK293T cells along with FLAG-tagged ISG15 to recapitulate ISGylation within the cells. These cells were also transfected with either the catalytically active or the catalytically inactive (active site cysteine-to-alanine substitution) PLP2 of three human coronaviruses, 229E, HKU1, and OC43, in addition to the PLP2 of MHV-A59. By performing Western blotting of the whole-cell lysates generated from the transfected cells, we were able to profile the deISGylase activity of the expressed PLP2s by assessing whether global cellular ISGylation levels were diminished. Our results demonstrated that the catalytically active MHV-A59 PLP2 had robust deISGylase activity (Fig. 1C, lane 2). This was also true for the catalytically active human coronaviruses 229E and OC43 (Fig. 1C, lanes 4 and 8). However, the human coronavirus HKU1 had little discernible deISGylase activity (Fig. 1C, lane 6) when compared to its catalytically inactive version (Fig. 1C, lane 7).

To corroborate these findings, we next examined whether HKU1 PLP2 was able to deISGylate MDA5, which is known to be deISGylated by SARS-CoV-2 PLpro. To test this, we used the same overexpression system described above but also overexpressed the GST-fused 2CARD domain of MDA5, the domain that is known to be ISGylated (21). By pulling down GST-MDA5 2CARD in the presence of different coronavirus PLP2 enzymes and probing for ISGylation via Western blotting, we determined that, again, HKU1 PLP2 exhibited minimal deISGylase activity (Fig. 1D, compare lanes 6 and 7), whereas the catalytically active PLPs of the other viruses were able to robustly deISGylate GST-MDA5 2CARD. Together, these results indicate that HKU1 PLP2 has markedly less deISGylase activity than MHV PLP2.

### Generating and characterizing the MHV-HP2 chimeric virus

**Fig 2:**
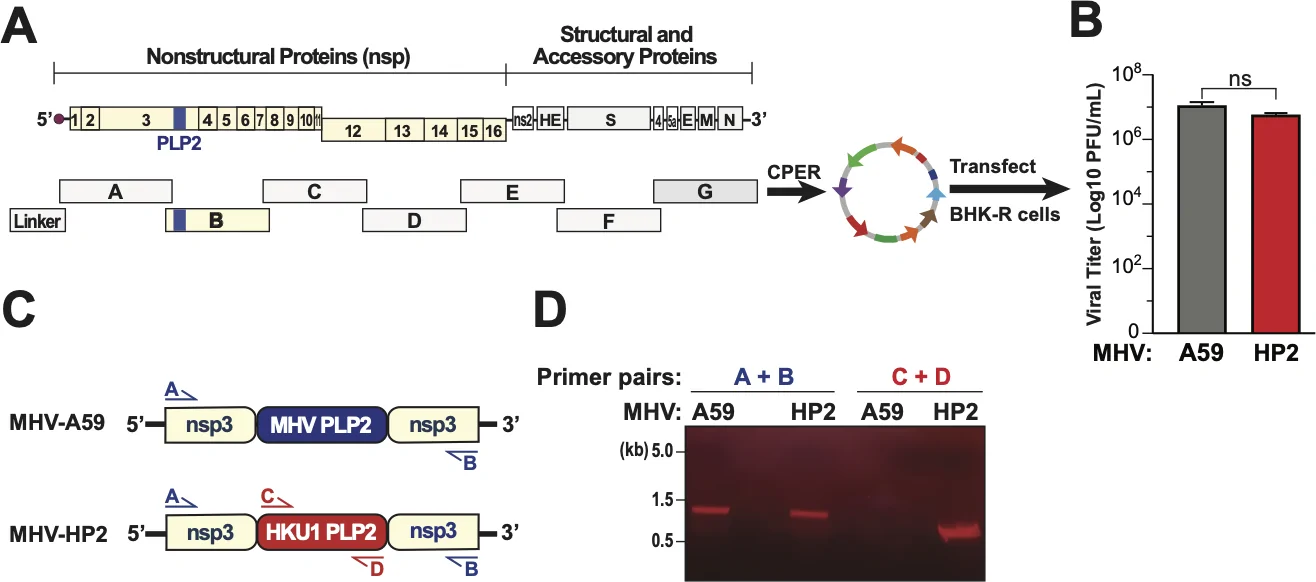
Generation of MHV-HP2 chimeric virus. (**A**) Schematic diagram of the CPER-based reverse genetics platform for generating recombinant MHV-HP2 virus. (**B**) BHK-R cells were infected with MHV-A59 or MHV-HP2 at an MOI of 0.1, and viral titers were measured at 24 hpi via plaque assay performed on 17Cl-1 cells. Results are representative of two independent experiments assayed in triplicate; results are represented as mean (± SD). Statistical analysis was performed via Welch’s *t*-test. (**C**) Two sets of primers were used for PCR amplification of MHV-nsp3 (primers A and B) and HKU1 PLP2 (primers C and D). (**D**) Electrophoresis of amplified PCR products on an agarose gel stained with ethidium bromide.

Given the remarkable structural homology of HKU1 PLP2 and MHV PLP2 (Fig. 1B), we hypothesized that we could replace the open reading frame (ORF) of the MHV PLP2 with the ORF of the HKU1 PLP2 to generate a chimeric virus with attenuated deISGylase activity, designated MHV-HP2. To generate the chimeric MHV-HP2, we used a reverse genetics system that relied on an optimized circular polymerase extension reaction (CPER) (Fig. 2A) (31, 32). After replacing the MHV PLP2 ORF with the HKU1 PLP2 ORF and generating amplicons spanning the length of the coronavirus genome, the resulting amplicons were subjected to CPER and transfected into baby hamster kidney cells expressing the MHV receptor (BHK-R). Once the cells showed signs of cytopathic effect, the supernatants were harvested, infectious virus was plaque-purified, passaged to generate a high-titer stock, and infectious titer was measured via plaque assay. We observed that MHV-HP2 replicated to high titer in BHK-R cells, generating equivalent titers to MHV-A59 by 24 hours post-infection (hpi) (Fig. 2B) despite exhibiting a smaller plaque phenotype (Fig. S1A and B). To determine if the chimeric stock virus contained the HKU1 PLP2 ORF, RNA was extracted from the viral supernatant and converted into cDNA. Then, the cDNA was amplified with primers specific to the broader nsp3 region (Fig. 2C, primers A and B) or with primers specific to HKU1 PLP2 (Fig. 2C, primers C and D). As revealed by gel electrophoresis, only the viral RNA isolated from MHV-HP2 contained a region identified by primers C and D, which are specific for HKU1 PLP2 (Fig. 2D). The full sequence of the amplified region was subsequently verified by Sanger sequencing.

**Fig 3:**
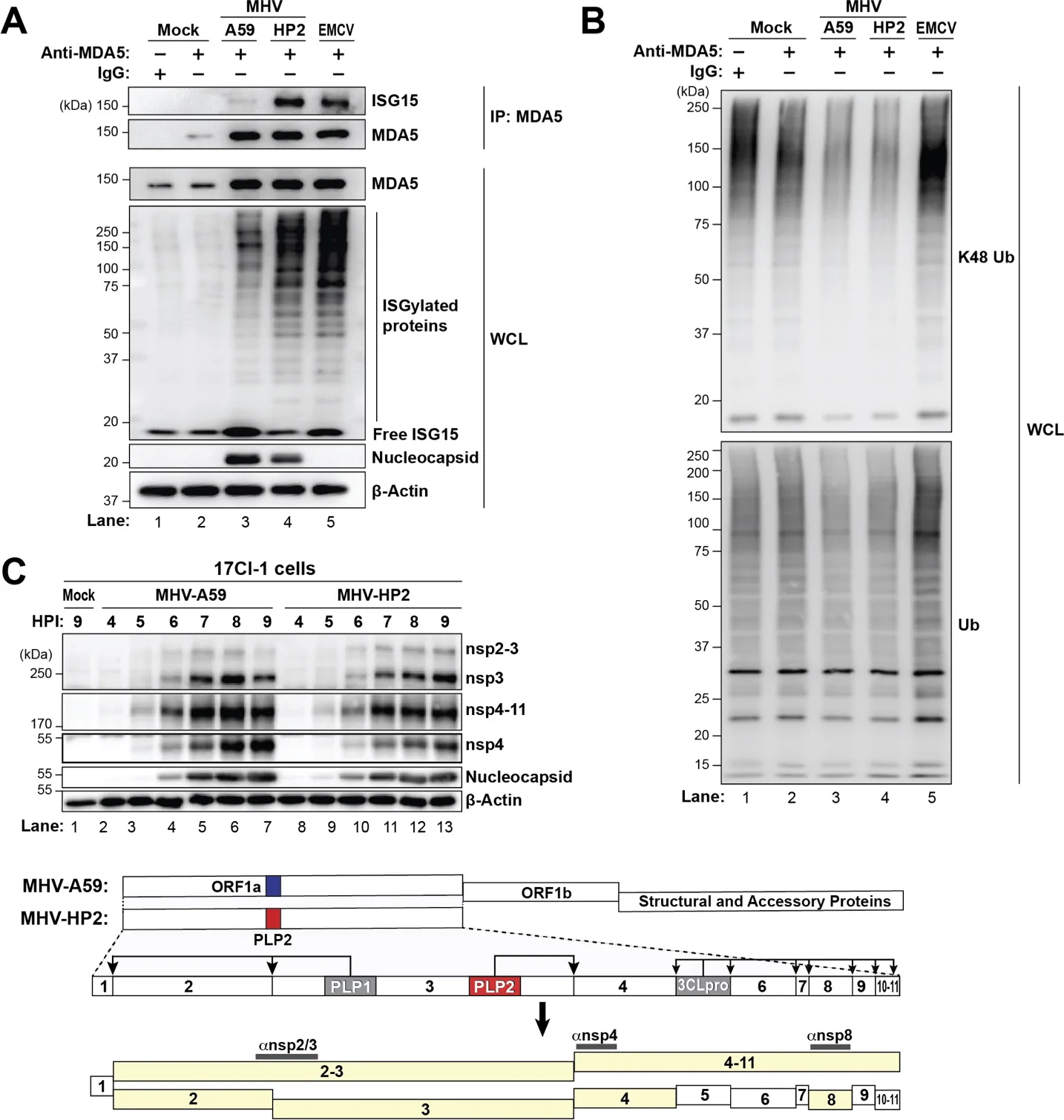
MHV-HP2 is impaired in deISGylase activity, retains DUB activity, and has a proteolytic processing delay. (**A and B**) Wild-type (WT) C57BL/6 BMDMs were either mock-infected or infected with the indicated viruses at an MOI of 1 for 8 hours. WCLs were generated and subjected to immunoprecipitation (IP) with either anti-MDA5 or isotype control IgG followed by Western blot with anti-ISG15. IPs (**A**, top panel) and WCLs (**A,** bottom panel, and **B**) were further analyzed via Western blot for the indicated proteins. (**C**) Top: 17Cl-1 cells were infected with either MHV-A59 or MHV-HP2 at an MOI of 1 or incubated with media alone (Mock). At each indicated time point, cells were harvested and WCLs were generated. Accumulation of polyprotein cleavage products was assessed via Western blotting with the indicated antibodies. Bottom: schematic of coronavirus genome with ORF1a cleavage products and their corresponding target antibodies. All the results are representative of two independent experiments.

We next investigated the three different PLP activities of the rescued virus. To assess the deISGylase activity of the two viruses, we infected bone marrow-derived macrophages (BMDMs), which are IFN-responsive and are thus capable of robust ISGylation, with either MHV-A59 or MHV-HP2. We profiled the ISGylation status of MDA5 by immunoprecipitating endogenous MDA5 from infected BMDMs. As a control, we also infected BMDMs with the picornavirus encephalomyocarditis virus (EMCV), a virus that is known to lack deISGylase activity. We observed that MDA5 ISGylation was very low in uninfected cells and MHV-A59-infected cells (Fig. 3A, lanes 2 and 3, top panel). In contrast, MHV-HP2 and EMCV infection resulted in robust ISGylation of MDA5, signifying that the deISGylase activity of MHV-HP2 directed against MDA5 was indeed compromised (Fig. 3A, lanes 4 and 5, top panel). Furthermore, analysis of WCLs revealed higher amounts of ISGylation and lower amounts of free unconjugated ISG15 in cells infected with MHV-HP2 compared to cells infected with MHV-A59, indicating a global defect in deISGylation (Fig. 3A, lanes 4 and 3, bottom panel).

Next, we determined whether the chimeric virus had intact deubiquitinase (DUB) activity. To assess the DUB activity of the chimeric virus, we took the WCLs from the above experiment and measured global cellular ubiquitination. Given previous work showing that the PLpro enzymes from several coronaviruses have a preference for K48-linked ubiquitin chains (16, 18, 33), we focused our attention on global K48-linked ubiquitination (Fig. 3B, top panel) along with total ubiquitination (Fig. 3B, bottom panel). We found that, as compared to mock infection, MHV-HP2 and MHV-A59 infection diminished K48-linked ubiquitination and total ubiquitination to a similar extent (Fig. 3B, lanes 3 and 4), indicating comparable DUB activity. Of note, MHV-A59- and MHV-HP2-infected cells demonstrated lower levels of ubiquitination when compared to EMCV-infected cells (Fig. 3B, lane 5), which is consistent with the expression of an active DUB by MHV.

Finally, we assessed PLP2 proteolytic function. PLP2 is responsible for cleaving the junction between nsp3 and nsp4 in ORF1a (Fig. 3C, bottom panel). By measuring the accumulation of nsp3 and nsp4 over time in murine fibroblast 17Cl-1 cells, we determined that MHV-HP2 PLP2 was able to process the ORF1a polypeptide, but with lower efficiency compared to MHV-A59 PLP2 (compare accumulation of nsp3 and nsp4 at 7 and 8 hpi) (Fig. 3C, top panel; quantified in Fig. S2). Together, these data provide evidence that MHV-HP2 is a replication-competent murine coronavirus with impaired deISGylase activity, retained DUB activity, and delayed protease activity.

### Evaluating the impact of MHV-HP2 proteolytic processing delay on viral replication

**Fig 4:**
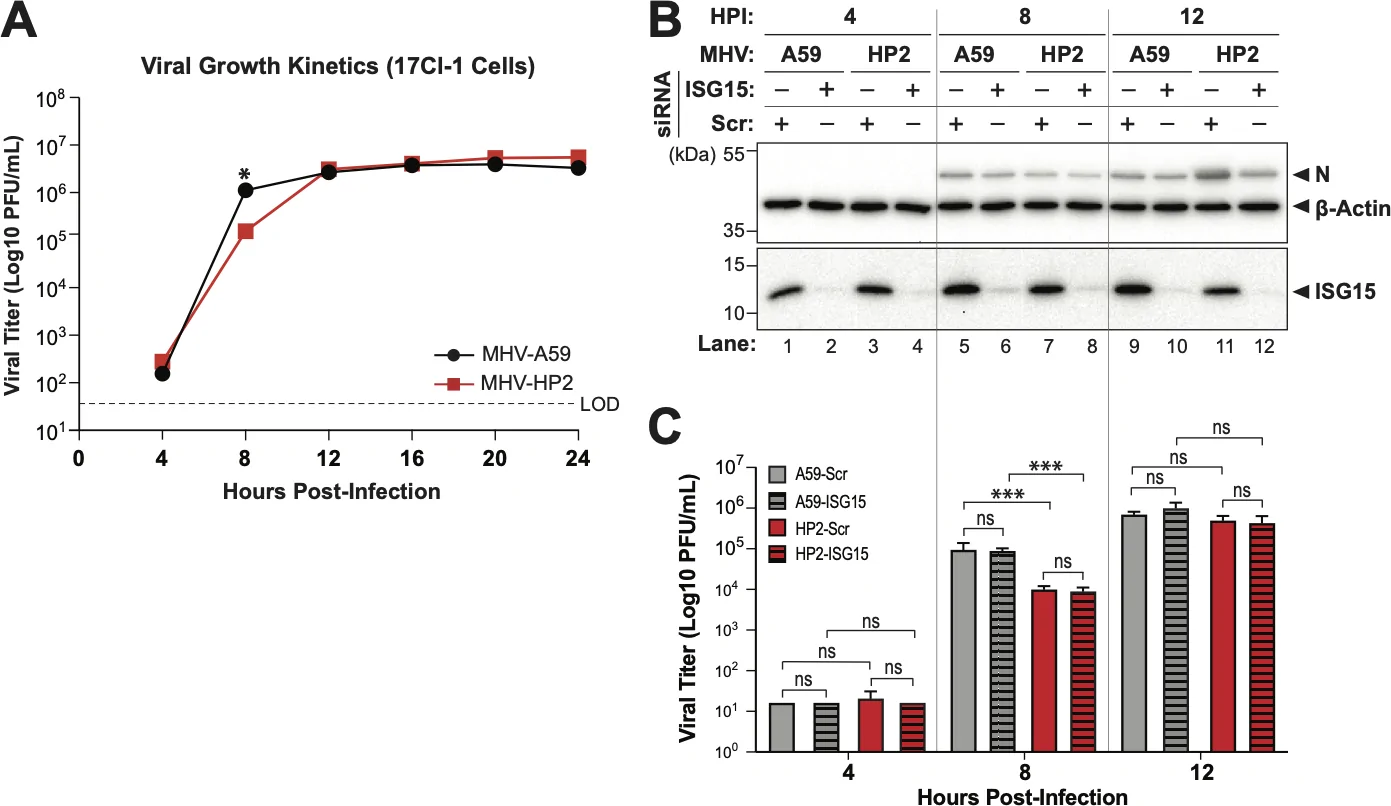
MHV-HP2 has a transient replication delay that is not rescued by ISG15 silencing. (**A**) 17Cl-1 cells were infected either with MHV-A59 or MHV-HP2 at an MOI of 0.1. At the indicated times, cell-free supernatants were harvested and subjected to plaque assay on 17Cl-1 cells. Data are presented as the means (± SD) of technical triplicates and are representative of *n* = 2 biological replicates. Data were analyzed via independent-sample *t*-tests by time point and confirmed using two-way ANOVA and a “within time analysis.” To account for concerns of multiple testing, α = 0.01 was used as the cut-off to declare significance. Significance threshold: (*) *P* < 0.01, (**) *P* < 0.001; there was no significant difference in titer at all other time points. (**B and C**) 17Cl-1 cells were transfected with either non-targeting or ISG15-targeting siRNAs (siRNA-Scr or siRNA-ISG15, respectively) 2 days prior to infection either with MHV-A59 or MHV-HP2 at an MOI of 0.1. At the indicated times, cell lysates were harvested for Western blot analysis (**B**) and cell-free supernatants were collected for plaque assay assessment (**C**). Data are representative of *n* = 2 biological replicates, with plaque-forming unit (PFU) data depicting means (± SD) of technical triplicates. Plaque assay data were analyzed via one-way ANOVA fits on the log10-virus-titer data separately for each of the time points, using Tukey HSD-adjusted F and *t*-tests to perform the depicted pairwise tests. (*) *P* < 0.01; (**) *P* < 0.01; (***) *P* < 0.001; (****) *P* < 0.0001; (ns) not significant. HPI, hours post-infection; LOD, limit of detection.

Given our observation that MHV-HP2 has a detectable proteolytic processing delay at 8 hpi, we investigated whether this would also translate to a replication delay. We characterized the replication kinetics of MHV-HP2 vs. MHV-A59 in the highly permissive immortalized cell line, 17Cl-1. These cells lack the ability to initiate a robust type I IFN response during MHV-A59 infection, making them highly permissive to virus replication (34). We observed that in 17Cl-1 cells, MHV-HP2 exhibited a reproducible growth delay in the exponential phase at 8 hpi; however, this difference was no longer detectable by 12 hpi, where MHV-HP2 titers were equivalent to those of MHV-A59 (Fig. 4A). We hypothesized that the early delay in replication may be due to either the loss of deISGylase activity or an alteration in the efficiency of proteolytic processing introduced by switching MHV PLP2 with PLP2 of HKU1.

To test whether proteolytic processing inefficiency or loss of viral deISGylase activity contributed to the replication delay, we used siRNA to knock down ISG15 in two cell types, 17Cl-1 cells and the mouse astrocytoma cell line DBT, and assessed for rescue of viral replication. We observed that 17Cl-1 and DBT cells expressed ISG15 and that siRNA against ISG15, but not a scramble control siRNA, resulted in a dramatic reduction in endogenous ISG15 detected by Western blot (Fig. S3). Interestingly, we observed that even in the nearly complete absence of ISG15 in the knockdown cells, MHV-HP2 titers were not rescued compared to MHV-A59 in 17Cl-1 cells (Fig. 4B and C) or DBT cells (Fig. S4A and B).

Given that the above findings were in cells that lacked the ability to generate a robust type I IFN response, we next wanted to determine if MHV-HP2 was also attenuated in IFN-responsive cells, and if an intact innate immune response contributes to its attenuation. We infected mouse BMDMs with either MHV-A59 or MHV-HP2 at low and high multiplicities of infection (MOIs) and found that MHV-HP2 is persistently attenuated in IFN-responsive cells (Fig. S5A and B). To determine if this attenuation is due to increased activation of the type I IFN response, we measured levels of IFN-α and IFN-β at multiple time points post-infection. We found that MHV-HP2 consistently triggered lower levels of type I IFN (Fig. S5C and D), indicating that innate immune signaling is not responsible for decreased viral replication. Similarly, infection of IFN⍺ receptor-deficient (IFNAR^−/−^) BMDMs with MHV-A59 or MHV-HP2 revealed that MHV-HP2 replication could not be rescued in the absence of type I IFN signaling (Fig. S5E). Importantly, infection of ISG15^−/−^ BMDMs revealed that MHV-HP2 replication could not be rescued in the complete absence of ISG15 either (Fig. S5F). These findings illustrate that MHV-HP2 attenuation is independent of innate immune signaling.

Together, our results are consistent with the idea that the proteolytic processing delay in MHV-HP2, rather than diminished deISGylase activity, strongly contributes to the transient delay observed in viral replication in cell culture.

### MHV-HP2 demonstrates earlier viral clearance and exhibits reduced pathogenesis and inflammation *in vivo*

**Fig 5:**
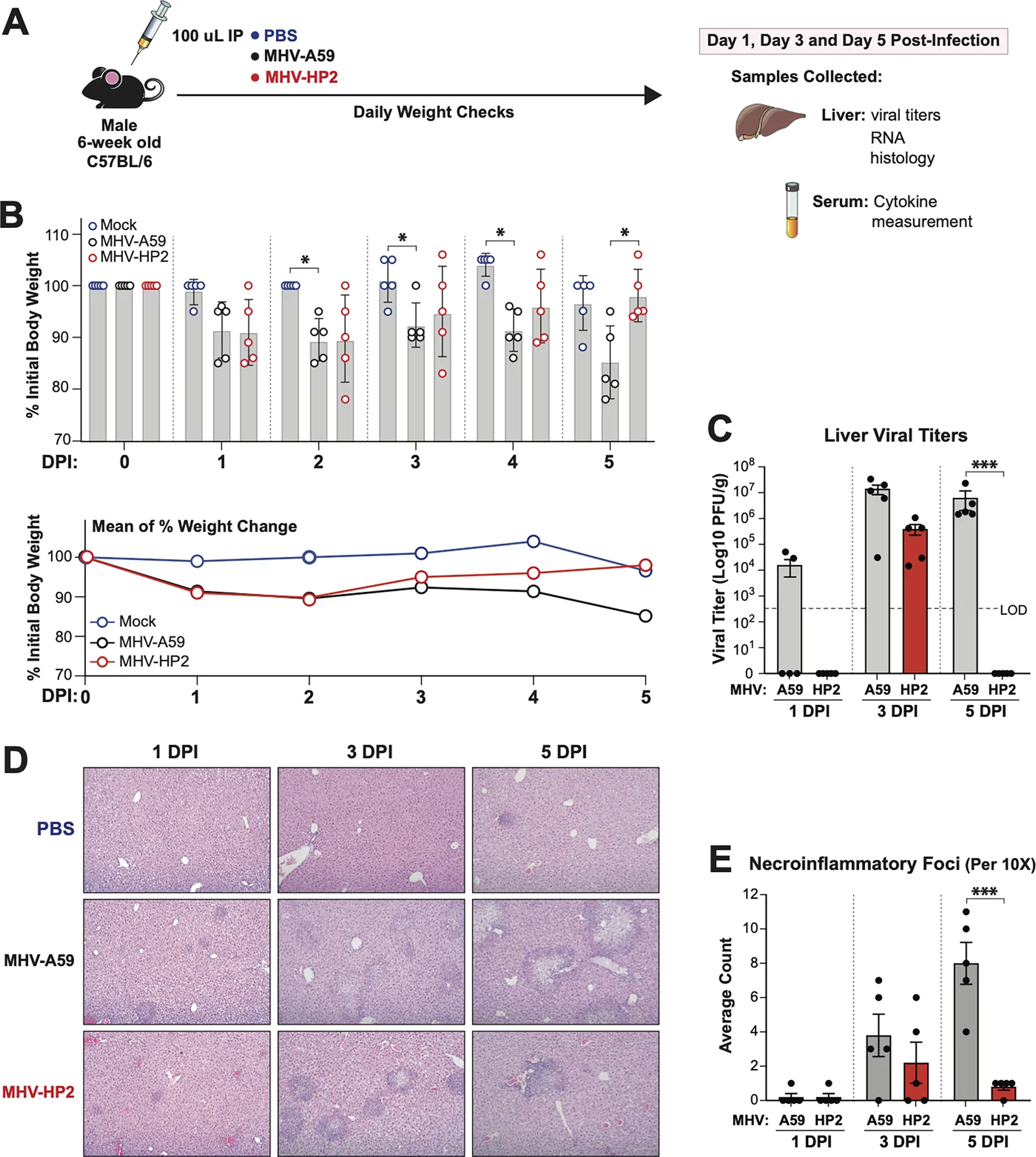
MHV-HP2 is attenuated in replication and pathogenesis *in vivo*. Six-week-old WT C57BL/6 mice were intraperitoneally mock-treated with PBS or injected with 6 × 10^4^ PFU of MHV-A59 or MHV-HP2 (*n* = 5 per cohort). (**A**) Experimental outline for *in vivo* MHV-A59 and MHV-HP2 infection. (**B**) Top: Percent weight change of uninfected, MHV-A59- and MHV-HP2-infected mice at 0–5 dpi. Statistical significance was calculated using Welch-adjusted one-way ANOVA tests; means were subsequently compared using the Games-Howell (nonparametric) multiple comparison procedure. Bottom: Mean weight change over 5 days of uninfected mice and mice infected with MHV-A59 or MHV-HP2. (**C**) Liver tissue homogenates were generated on the indicated day post-infection. Viral titers were measured via plaque assay in 17Cl-1 cells at 1, 3, and 5 dpi and normalized to grams of liver tissue. Data are represented as the mean (± SEM) of five mice per group. Statistical significance was calculated via Mann-Whitney *U*-test (two-tailed). (**D**) Whole livers were excised, fixed in formalin, stained with H&E, and imaged. Representative images (10× magnification) are shown here. (**E**) The necroinflammatory foci per 10× field were counted from 10 fields per group, with the number of foci indicated for each mouse. Data are represented as the mean (± SEM) of five mice per group. Statistical significance was calculated via ordinary one-way ANOVA. Significance thresholds: (*) *P* < 0.05; (**) *P* < 0.01; (***) *P* < 0.001; (ns) not significant. dpi, days post-infection. LOD, limit of detection. These results are representative of three independent experiments.

We next sought to investigate whether the net impact of our observed PLP2 phenotypes (Fig. 3) influences *in vivo* replication and pathogenesis. We intraperitoneally inoculated 6-week-old male mice with either MHV-A59 or MHV-HP2, recorded body weights of the mice daily, and harvested liver and serum for analysis (Fig. 5A). We observed that mice infected with MHV-A59 lost a significant amount of weight compared to uninfected mice as early as 2 days post-infection (dpi), and the weight loss was significant out to 4 dpi (Fig. 5B, top). For mice infected with MHV-HP2, there was a transient weight loss with recovery as assessed by the mean percent weight change (Fig. 5B, bottom). Importantly, at 5 dpi, mice infected with MHV-A59 had lost significantly more weight compared to mice infected with MHV-HP2 (Fig. 5B, top). We observed that all mice infected with MHV-HP2 were able to clear detectable levels of infectious virus from the liver by day 5, whereas mice infected with MHV-A59 showed evidence of prolonged virus replication (Fig. 5C).

We compared pathological changes in the livers of mice injected intraperitoneally with either PBS (mock-infected group), MHV-A59, or MHV-HP2 (infected groups). We observed that mice injected with MHV-A59 exhibited hepatitis characterized by time-dependent hepatocyte damage with increasing necroinflammatory foci that began coalescing as infection proceeded. The PBS-injected control mice experienced minimal histologic changes. The mice injected with MHV-HP2 showed intermediate histologic changes with peak necroinflammatory foci at 3 dpi that were less than those seen in MHV-A59-infected mice. Furthermore, the necroinflammatory foci in MHV-HP2-infected mice trended down by day 5, which contrasts with the levels detected in MHV-A59-infected mice (Fig. 5D and E).

**Fig 6:**
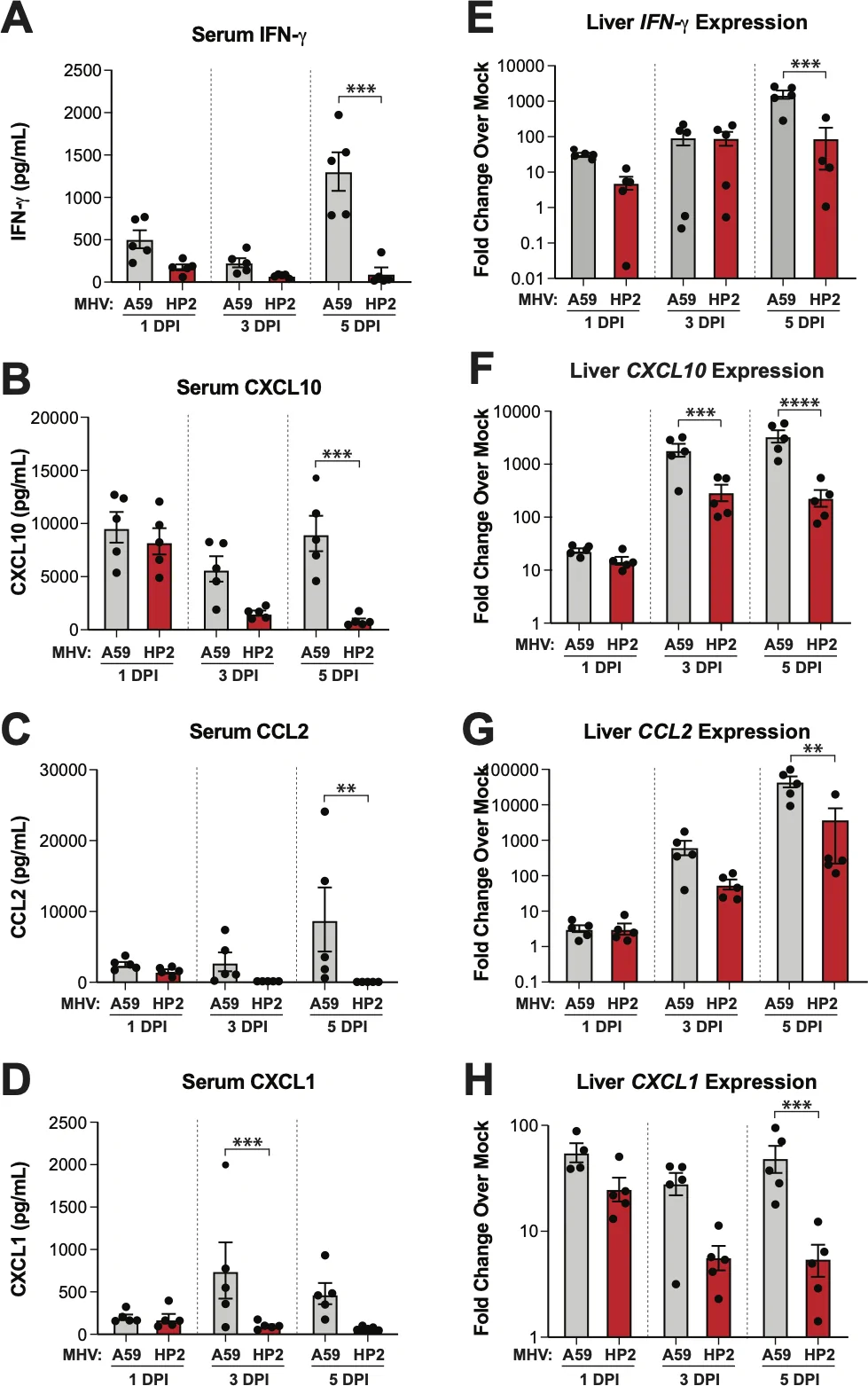
MHV-HP2 *in vivo* infection is associated with earlier resolution of inflammation. Six-week-old WT C57BL/6 mice were intraperitoneally mock-treated with PBS or injected with 6 × 10^4^ PFU MHV-A59 or MHV-HP2 (*n* = 5 per cohort). (**A–D**) Sera were collected from mice on the indicated day post-infection, heat-inactivated, and profiled for the expression of IFN-γ (**A**), CXCL10 (**B**), CCL2 (**C**), and CXCL1 (**D**) via CBA. Data are represented as the mean (± SEM) of 5 mice, and statistical significance was calculated via ordinary one-way ANOVA (*P* < 0.05). (**E–H**) Livers of infected mice were harvested in TRIzol at 1, 3, and 5 dpi, and RNA was isolated from ~150 mg of tissue through chloroform extraction, converted to cDNA, and analyzed via qPCR for *IFN-γ* (**E**), *CXCL10* (**F**), *CCL2* (**G**), and *CXCL1* (**H**). Results were normalized to 18S rRNA levels and expressed as fold change over mock-infected mice (2^-ΔΔCt^). Data are presented as the mean (± SEM) of five mice per group, and statistical significance was calculated via ordinary one-way ANOVA. Significance thresholds: (*) *P* < 0.05; (**) *P* < 0.01; (***) *P* < 0.001; (****) *P* < 0.0001; (ns) not significant. dpi, days post-infection.

We hypothesized that viral clearance would result in earlier resolution of virally-induced inflammation. To test this hypothesis, we measured the serum cytokine response to viral infection using a commercially available 13-plex cytometric bead array (CBA). We also measured the corresponding levels of gene expression in the liver over the course of 5 days. Of the 13 cytokines assessed in the CBA, we found that mice infected with MHV-A59 had the highest levels of serum IFN-γ, CXCL10, CCL2, and CXCL1 as the infection progressed through 5 dpi (Fig. 6A through D). However, mice infected with MHV-HP2 demonstrated a decrease in these same cytokines as early as 3 dpi (Fig. 6A through D). At the transcription level, levels of CXCL10, CCL2, and CXCL1 had significantly dropped in the liver of mice infected with MHV-HP2 by 5 dpi, while IFN-γ levels trended lower (Fig. 6E through H). Other cytokines, such as IL-1β, IL-6, TNF-α, and IFN-α, also showed higher levels of induction during MHV-A59 infection compared to MHV-HP2 infection (Fig. S6A through D).

Together, these data indicate that MHV-HP2, with diminished deISGylase activity and altered proteolytic activity, exhibits attenuated viral replication accompanied by a diminished host inflammatory response, in contrast to the deISGylase-competent, proteolytically efficient MHV-A59. Overall, these results document the *in vivo* replication and pathogenesis of a murine coronavirus with impaired deISGylase activity and delayed proteolytic activity.

## DISCUSSION

In this study, we sought to investigate the role of deISGylase activity in coronavirus replication and pathogenesis. Our approach was to generate a murine coronavirus lacking deISGylase activity, designated MHV-HP2. We leveraged recently published data revealing that the PLP2 of the human coronavirus HKU1 has the lowest deISGylase activity of the human coronavirus PLPs that were evaluated (28). We capitalized on the sequence and structural homology of HKU1 PLP2 to MHV-A59 PLP2 (Fig. 1A and B) and swapped HKU1 PLP2 into the context of MHV-A59. This allowed us to recover an infectious murine coronavirus that replicated to a high titer in cell culture (Fig. 2B) and lacked deISGylase activity (Fig. 3A).

Unexpectedly, detailed kinetic studies revealed that MHV-HP2 protease activity was also impaired at early time points (Fig. 3C, top panel). We observed that the delayed proteolytic activity coincided with a reproducible delay in the early replication of MHV-HP2 in highly permissive cells, which was unable to be rescued by ISG15 knockdown (Fig. 4; Fig. S4 and S5F). These results indicate that impaired proteolytic processing was likely responsible for the transient delay in MHV-HP2 replication. Accordingly, given the impaired proteolytic activity of MHV-HP2, the observed *in vivo* differences in pathogenesis are likely a combination of loss of deISGylase activity and delayed proteolytic activity. In addition to delayed proteolytic activity, we cannot exclude the possibility that replacement of the PLP2 coding region introduced deleterious changes in secondary or tertiary structure of viral RNA, which may impact replication or transcription of MHV-HP2 RNA. Overall, our work highlights the challenge of separating the three enzymatic activities mediated by coronavirus papain-like proteases and that kinetic studies are essential for elucidating the effect of PLP mutations on the replication of the virus.

Future studies generating point mutations that specifically ablate deISGylase versus protease activity are needed to fully elucidate the contributions of each activity during viral infection and pathogenesis. Previous studies have illuminated the challenges of parsing out the multifunctionality of coronavirus PLP2/PLpro. Békés and colleagues identified residues in SARS-CoV PLpro that were specific for ISG15 or ubiquitin (18). Deng and colleagues showed *in vitro* that the R1803A mutation in MHV-A59 resulted in a deISGylase-deficient phenotype but were unable to recapitulate the phenotype with the rescued virus (35). Shin et al. and Wydorski et al. identified residues in SARS-CoV-2 PLpro that interacted specifically with ubiquitin or ISG15 (8, 33). Van Huizen and colleagues showed that abrogating deISGylase activity in SARS-CoV-2 also negatively impacted viral polyprotein processing, resulting in nonviable viruses (19). None of these attempts resulted in generation of a deISGylase-deficient virus, highlighting the challenge of decoupling PLP-mediated deISGylase activity from proteolytic processing activity.

Importantly, recent work by Zhu and colleagues describes generation of a mutant SARS-CoV-2 with two amino acid substitutions in PLpro resulting in ablated PLpro deISGylase activity (36). This mutant virus is unable to deISGylate MDA5 and is attenuated in viral replication and pathogenesis. This study also identified multiple host substrates involved in metabolism that are deISGylated by SARS-CoV-2 PLpro, which contributes to the growing body of work elucidating the role of viral deISGylation in regulating diverse host cellular pathways. For example, recent evidence suggests that the host protein stimulator of IFN genes (STING) undergoes ISGylation to prevent its ubiquitin-mediated degradation, and that SARS-CoV-2 PLpro counteracts this ISGylation (37). While these data relied on overexpression experiments to demonstrate protein ISGylation, it is possible that STING represents a target in coronavirus-infected cells as a means of suppressing STING-induced IFN response. Additionally, cyclic GMP-AMP synthase (cGAS) has been shown to be ISGylated in the context of herpes simplex virus-1 infection, which allows the protein to oligomerize and induce the expression of many virus-stimulated genes and ISGs (38, 39). It’s possible that coronavirus PLP activity can catalyze the deISGylation of cGAS, allowing for augmented virus replication. In the case of cGAS, viral RNA would not be the relevant pathogen-associated molecular pattern; rather, recent evidence suggests that cGAS recognizes mitochondrial DNA produced as a consequence of coronavirus infection (40).

In addition to host proteins, coronaviral proteins can also be ISGylated to promote an antiviral state. For example, SARS-CoV-2 nucleocapsid (N) protein contains multiple lysine residues that are sites for ISGylation, and SARS-CoV-2 PLpro catalytically removes ISG15 from ISGylated N proteins (41, 42). These studies suggest that ISGylation of N protein prevents its oligomerization, which disrupts viral replication (41, 42). While this has not yet been shown for MHV-A59 N protein, it is possible that one contributor to MHV-HP2 attenuation in IFN-responsive cells is the inability of the MHV-HP2 PLP2 to remove ISG15 from the N protein, preventing proper replication. Future work is needed to investigate how (de)ISGylation of coronavirus proteins affects viral replication.

In summary, our study investigates the critical role that coronavirus PLP enzymatic activities play in promoting viral pathogenesis and further illustrates that targeting PLP activity can be an effective strategy for mitigating coronavirus disease.

## MATERIALS AND METHODS

### Cells and viruses

Human embryonic kidney (HEK) 293T cells were purchased from ATCC (CRL-11268). Baby hamster kidney cells expressing the MHV receptor (BHK-R cells) were a gift from Mark Denison (Vanderbilt University Medical Center, Nashville, TN) and were maintained in DMEM + 10% fetal bovine serum (FBS). Murine fibroblast 17Cl-1 cells were cultured in DMEM + 5% FBS, and murine astrocytoma delayed brain tumor (DBT) cells were maintained in MEM + 5% FBS. For generation of BMDMs, tibias and femurs from C57BL/6 mice were isolated from euthanized mice. Bone marrow was collected and differentiated into BMDMs as described previously (43). MHV-A59 (GenBank accession no. AY910861) was generated previously via reverse genetics, and its full genome was sequenced (43). The EMCV-EMC strain was purchased from ATCC (VR-129B) and propagated in HEK293T cells.

### Animals

Five-week-old male C57BL/6J (Cat #00064) mice were purchased from the Jackson Laboratory (Bar Harbor, ME, USA) and acclimated for 1 week in the Animal Biosafety Level 2 in the Comparative Biomedical Facilities (CMF) at Loyola University Health Sciences Campus prior to any inoculations. Animals were housed five per cage with food and water provided *ad libitum*, with a 12/12 light/dark cycle.

### Mouse infections

Male 6-week-old mice were inoculated via intraperitoneal injection using a 26-gauge needle (BD PrecisionGlide, Cat #305110, Franklin Lakes, NJ, USA) with 6 × 10^4^ PFUs of either MHV-A59, MHV-HP2, or mock in 100 µL of 1× calcium-, magnesium-free PBS (1× CMF-PBS) made from a 10× stock (Fisher Scientific, Cat #70-011-044). Mouse weights were monitored daily over the course of 6 days, starting with day 0 until 5 dpi, with three harvest time points at 1, 3, and 5 dpi. Infections were repeated in three separate experiments.

### Mouse euthanasia and tissue harvest

Mice were euthanized at 1, 3, and 5 dpi by inhalation of isoflurane (Isospire, NDC Code: 17033-091-25, Overland Park, KS, USA) via drop method, followed by thoracotomy, with livers harvested at each time point. An estimated 120 mg of liver was collected and placed in 1.7 mL Eppendorf tubes (Fisher Scientific, Cat #NC9448938) with 1 mL of 10% buffered formalin (Sigma-Aldrich, Cat #HT501128, St. Louis, MO, USA) for histology analysis. A second liver sample of ~50 mg was collected and placed in 2 mL capped microcentrifuge tubes (Fisherbrand, Cat #02-682-558) for plaque assays.

### Tissue homogenates and TRIzol RNA extraction

Incomplete DMEM without FBS was used to make 10% tissue homogenates (wt/vol) of harvested liver. Livers were lysed using 3 mm glass beads (MilliporeSigma C959A68). Tissues were homogenized with a tissue lyser (Fisherbrand 15-340-163) used at 6.0 m/s for 30 seconds. After lysing, tubes were centrifuged at 450 × *g* at 4°C for 5 minutes, and 100 µL of each supernatant was collected and stored at −80°C for infectious virus titration by plaque assay. Serum was collected for cytokine measurement using a commercial bead array kit (BioLegend 740622).

### Bulk cellular deISGylation assay

HEK293T cells were seeded into six-well plates and transfected using *Trans*IT-LT1 transfection reagent (MIR 2304) with plasmid DNA encoding HA-Ube1L, FLAG-UbcH8, and FLAG-ISG15 (21), and a catalytically active or catalytically inactive (C/A) V5-tagged PLP2 from either MHV-A59, 229E, HKU1, or OC43. After transfection, the cells were allowed to grow overnight at 37°C. The cells were then solubilized in RIPA buffer, further lysed by sonicating for 10 seconds at 50% amplitude, and cleared at 18,000 × *g* for 10 minutes at 4°C. Cleared lysates were dissolved 1:1 in 2× Laemmli sample buffer. Bulk deISGylation was assessed by analyzing the samples via Western blot and detecting ISGylation with anti-FLAG (M2, 1:2,000 dilution) antibody. Expression of other transfected proteins was validated using anti-HA (1:2,000 dilution), anti-FLAG (1:2,000 dilution), and anti-V5 (1:5,000 dilution) antibodies.

### GST pulldown assay

HEK293T cells were seeded into six-well plates and transfected as described for the bulk cellular deISGylation assay, and additionally with plasmid DNA encoding GST-MDA5 2CARD. Cells were allowed to grow overnight at 37°C, and then the cells were solubilized in 300 µL RIPA buffer (+1 mM DTT), lysed by sonicating for 10 seconds at 50% amplitude, and cleared by centrifugation at 18,000 × *g* for 10 minutes at 4°C. Equal volumes of 2× Laemmli sample buffer were added to 50 µL of cleared lysate to generate WCLs. The remainder of the lysate was diluted 1:1 with GST equilibration buffer (125 mM Tris-HCl, pH 7.4, 150 mM NaCl, 1 mM EDTA, 1 mM DTT, and 1× PI cocktail [GoldBio]). Glutathione-agarose beads were washed with equilibration buffer and resuspended in 100 µL of equilibration buffer per sample. A total of 125 µL of beads was then added to the diluted lysates, vortexed briefly, and incubated on a rotator for 1–2 hours at 4°C. The beads were collected on a magnetic stand, and supernatants were aspirated. Beads were washed twice with 500 µL of equilibration buffer, and protein was eluted from beads using 1× Laemmli sample buffer. Beads were boiled for 10 minutes, and the resulting elution was run on SDS-PAGE and analyzed via Western blot for 2CARD pulldown and ISGylation with anti-GST and anti-FLAG, respectively. WCLs were also analyzed via Western blot for protein expression using anti-GST, anti-FLAG, anti-HA, and anti-V5 antibodies.

### Generation of MHV-HP2 chimeric virus with CPER

The seven-plasmid reverse-genetics platform of the MHV-A59 genome (GenBank AY910861.1) previously generated in our lab (32) was used as the foundation for the generation of the MHV-HP2 chimeric virus, which contains the PLP2 domain of the HKU1 virus in the background of MHV-A59. One of the plasmids, Plasmid B, contained the PLP2 domain of MHV-A59. To generate the MHV-HP2 virus, the MHV-A59 PLP2 ORF was removed via PCR amplification of Plasmid B with primers flanking the PLP2 ORF (forward primer: 5′ CCGGTTGTCTGGCTTGGCC 3′; reverse primer: 5′ GTGAGACGAAGGGCGAATTCTGC 3′). The resulting opened-up plasmid was combined in a Gibson reaction with a gBlock (IDT DNA) containing the HKU1 PLP2 ORF and 5′ and 3′ 19 bp overlaps with the opened-up Plasmid B, allowing for the generation of a modified Plasmid B with HKU1 PLP2 in the place of MHV-A59 PLP2. The modified Plasmid B was expanded and sequenced.

The other six plasmids, the newly modified Plasmid B, and a linker plasmid containing the CMV enhancer and promoter were PCR-amplified with primers that contained 19–21 nucleotide overlaps to generate amplicons that overlapped with adjacent viral amplicons. After PCR amplification, amplicons were gel-purified. For the generation of the final CPER product, 0.05 picomole of each amplicon was mixed with PrimeSTAR GXL DNA polymerase (Takara Bio, R050A). The resulting product was transfected into BHK-R cells using the transfection reagent TransIT-X2 (Mirus Bio, MIR 6004). Once a cytopathic effect was observed, cell-free supernatants were harvested, aliquoted, and stored at −80°C.

To generate viral stocks, BHK-R cells were seeded into six-well plates and infected with serial dilutions of the CPER-generated viral supernatants. Discrete plaques were identified, picked via aspiration using a pipette tip, and ejected into incomplete DMEM. The picked plaque was used as inoculum for BHK-R cells, and the cells were infected overnight. The resulting viral supernatant was collected and titered via plaque assay on 17Cl-1 cells. After viral titers were determined, BHK-R cells were seeded into T75 flasks and infected with MHV-HP2 at an MOI of 0.1. At peak cytopathic effect, viral supernatants were collected, spun down at 300 × *g* for 5 minutes, aliquoted, and frozen at −80°C. The resulting viral stock was titered via plaque assay on 17Cl-1 cells and used for all future experiments.

### PCR validation of MHV-HP2 genomic insert

Viral RNA was harvested from liver tissue homogenized in TRIzol reagent using chloroform phase separation. A total of 1 µg of isolated RNA was converted into cDNA using the RevertAid First Strand cDNA Synthesis Kit (Thermo Scientific, K1621). The cDNA template was diluted 1:10 in RNase-free water and amplified via PCR with primers specific to nsp3 (Fig. 2C, forward primer A: 5′ TTAAGTATTTTGAATGCCCGG 3′; reverse primer B: 5′ CAGACTCACAGTAATACTGC 3′) or HKU1 PLP2 (Fig. 2C, forward primer C: 5′ GCTAAGAAGATAGATGTTTTGC 3′; reverse primer D: 5′ AGCACAGATGTAAAAGTCTGGG 3′). Amplicons were then run on a 0.8% agarose DNA gel in 1× TAE buffer and stained with an ethidium bromide solution. Gels were imaged via UV light.

### Viral kinetics and plaque assays

Cells used in viral kinetic assays (as indicated in the figure legend) were plated in 12-well plates and allowed to grow overnight. Cells were infected in duplicate at an MOI of 0.1. At indicated times, cell-free supernatants were collected and frozen at −80°C. Viral titers were quantified via plaque assay on 17Cl-1 cells.

Plaque assays were performed as follows: 5 × 10^5^ 17Cl-1 cells were seeded into a six-well plate and allowed to grow overnight. Ten-fold serial dilutions of viral samples were prepared in cold, incomplete DMEM and used to inoculate the 17Cl-1 cells. After 1 hour of adsorption, cells were overlaid with 0.4% Noble agar-DMEM and incubated at 37°C for 48 hours. Cells were then fixed with 3.7% formaldehyde-PBS solution, stained with 0.1% crystal violet, and plaques were counted.

### Assessment of viral polyprotein processing

To determine if the replaced PLP2 domain of HKU1 virus in MHV-A59 background has any effect on the proteolytic processing function of PLP2, the kinetics of viral non-structural protein expression was performed using 17Cl-1 cells infected with either MHV-HP2 chimeric virus or MHV-A59 WT at an MOI of 0.1 for 4, 5, 6, 7, 8, and 9 hours. Cell lysates were prepared as previously described (44) in lysis buffer A (4% SDS, 3% DTT, 40% glycerol, and 0.0625 M Tris, pH 6.8). The samples were passed through a 22-gauge needle to shear chromosomal DNA and used directly or frozen at −80°C. The cell lysates were diluted in 2× Laemmli sample buffer (3% SDS, 5% β-mercaptoethanol, 10% glycerol, and 0.0625 M Tris, pH 6.8) with a ratio of 1:1 and incubated at 37°C for 30 minutes before loading onto SDS-PAGE. Three rabbit polyclonal antibodies, anti-nsp2/3, anti-nsp4, and anti-nsp8, were used in this study (45) at a dilution of 1:2,000 and followed with horseradish peroxidase (HRP)-donkey anti-rabbit monoclonal antibody at a dilution of 1:5,000.

### Viral deISGylation assay

For assaying endogenous MDA5 ISGylation, freshly differentiated BMDMs from C57BL/6 WT mice were plated 5 days post-differentiation into 10 cm dishes in complete BMDM media (RPMI medium supplemented with 10% FBS, 100 U/mL antibiotic-antimycotic [Gibco], 1% non-essential amino acids, and 1 mM sodium pyruvate) at a density of 5 × 10^6^ cells per dish. The next day, cells were infected with MHV-A59, MHV-HP2, or EMCV-EMC at an MOI of 1. At 8 hpi, the cells were scraped in 10 mL cold PBS, pelleted via centrifugation, and harvested in 500 µL Nonidet P-40 (NP-40) lysis buffer (50 mM HEPES [pH 7.2], 200 mM NaCl, 1% [vol/vol] NP-40, 5 mM EDTA, and 1× protease inhibitor [BioGold #GB-331-50]). Lysates were cleared via centrifugation at 16,000 × *g* and 4°C for 20 minutes. Next, 50 µL of the cleared lysate was added to 50 µL of 2× Laemmli sample buffer to generate WCL. The remainder was subjected to immunoprecipitation for the detection of endogenous MDA5.

For immunoprecipitation, the samples were incubated at 4°C for 16 hours using Protein G Dynabeads pre-conjugated with anti-MDA5 antibody (D74E4; Cell Signaling Technology) or rabbit IgG (DA1E; Cell Signaling Technology) as a control. The beads were extensively washed five times with NP-40 buffer. The proteins were eluted by heating with 1× Laemmli sample buffer at 95°C for 5 minutes. The immunoprecipitated samples and WCLs were resolved via SDS-PAGE gels and transferred onto polyvinylidene difluoride (PVDF) membranes (BioRad, #1620177). Membranes were incubated with primary and secondary antibodies, and protein signals were visualized using an ImageQuant LAS 4000 Chemiluminescent Image Analyzer (General Electric) as previously described (22).

### siRNA knockdown of ISG15

17CL-1 or DBT cells were transfected with RNAiMAX (Invitrogen, Cat #13778150, reverse transfection according to product manual) complexed with ON-TARGETplus siRNA for either Non-targeting Control Pool (Horizon, D-001810-10-05) or mouse ISG15 (Horizon, L-167630-00-0010, accession no. NM_015783) in six-well plates. At 24 hours post-transfection, cells were re-plated into 12-well plates. At 48 hours later, cells were infected with MHV-A59 or MHV-HP2 at an MOI of 0.1 for 1 hour in Opti-MEM. At 1 hpi, the inoculum was removed and cells were rinsed once with PBS and then replenished with fresh medium with 1% FBS. At the times indicated, cell-free supernatants were collected and frozen at −80°C for plaque assay assessment. The cells were rinsed once with PBS and harvested in 200 µL NP-40 lysis buffer (50 mM HEPES [pH 7.2], 200 mM NaCl, 1% [vol/vol] NP-40, 5 mM EDTA, and 1× protease inhibitor [BioGold #GB-331-50]). Lysates were clarified via centrifugation at 18,000 × *g* and 4°C for 15 minutes. Next, cleared lysate was mixed with 2× Laemmli sample buffer at a 1:1 ratio to generate WCL.

### RNA isolation and quantitative PCR analysis

BMDMs were lysed with 350 µL RLT lysis buffer, and RNA was extracted with the RNeasy Mini kit (Qiagen 74106). Next, 500 ng of total RNA was converted to cDNA using the RT^2^ First Strand Kit (Qiagen 330404), and quantitative PCR (qPCR) was performed using the RT^2^ SYBR Green qPCR Mastermix (Qiagen 330502). The following primers were used: mouse IFN-α11 (Qiagen PPM03050B-200); mouse IFN-β1 (Qiagen PPM03594C-200); mouse 18S rRNA (Qiagen PPM57735E-200); mouse IFN-γ (forward primer: 5′ ATGAACGCTACACACTGCATC, reverse primer: 5′ CCATCCTTTTGCCAGTTCCTC); mouse CXCL10 (forward primer: 5′ CCAAGTGCTGCCGTCATTTTC, reverse primer: 5′ GGCTCGCAGGGATGATTTCAA); mouse CCL2 (forward primer: 5′ TTAAAAACCTGGATCGGAACCAA, reverse primer: 5′ GCATTAGCTTCAGATTTACGGGT); mouse CXCL1 (forward primer: 5′ CTGGGATTCACCTCAAGAACATC, reverse primer: 5′ CAGGGTCAAGGCAAGCCTC); and MHV-A59 N gene (forward primer: 5′ AGCAGACTGCAACTACTCAACCCAACTC, reverse primer: 5′ GCAATAGGCACTCCTTGTCCTTCTGCA).

The cycling conditions were 95°C for 10 minutes followed by 40 cycles of 95°C for 15 seconds and 60°C for 60 seconds. A melt curve was generated by increasing the temperature from 65°C to 95°C by 0.5°C every 5 seconds. Gene expression was normalized to 18S rRNA (ΔCt). Fold change over mock (ΔΔCt) was calculated using 2^-ΔΔCt^.

### Western blotting

WCL samples were run on 12% Tris-glycine gels and transferred to PVDF membranes via semi-dry transfer at 25 V, 1.0 A for 30 minutes. Membranes were blocked for at least 1 hour in 5% milk in Tris-buffered saline solution with 0.1% (vol/vol) Tween 20 (TBST) and then incubated overnight with primary antibody diluted in 5% milk in TBST at 4°C. Membranes were subsequently incubated with HRP-conjugated secondary antibodies diluted in 5% milk in TBST at room temperature for 1 hour. Blots were developed using ECL Western blotting substrate (Thermo Fisher 32209) and visualized using the ProteinSimple FluorChem E System (Bio-Techne). Blots visualizing ISG15 were developed using SuperSignal West Femto Maximum Sensitivity Substrate (Thermo Scientific Cat #34095). The following antibodies were used in this study: mouse anti-HA.11 (1:2,000, BioLegend 901513), mouse anti-FLAG M2 (1:2,000, Sigma-Aldrich F1804), mouse anti-V5 (1:5,000, Invitrogen R960-25), mouse anti-ISG15 (1:1,000, Santa Cruz Biotechnology), mouse anti-β-actin (1:5,000, GenScript A00702-40), anti-MDA5 antibody (1:1,000, Cell Signaling Technology D74E4), anti-ubiquitin antibody (1:500, Santa Cruz P4D1), anti-K48-linkage-specific polyubiquitin antibody (1:1,000, Cell Signaling Technology D9D5), and mouse anti-MHV nucleocapsid (1:5,000, J.3.3, a gift from John Fleming at the University of Wisconsin-Madison). The following secondary antibodies were used: HRP-conjugated goat anti-mouse (1:5,000, SouthernBiotech 1010-05) and HRP-conjugated donkey anti-rabbit (1:5,000, Jackson ImmunoResearch 711-035-152).

### Statistical analysis

Statistical analyses were performed in Minitab (version 20), SAS (version 9.4), and RStudio (version 2025.09.2). All data are represented as mean ± SEM, unless stated otherwise. For viral growth kinetics plaque assay data, viral titers of MHV-A59 versus MHV-HP2 viruses were compared after first log_10_-transforming the data to stabilize variances and render the data normal. Statistical analyses for siRNA data were performed in SAS. For these viral growth kinetics plaque assay data, virus titer of MHV-A59 versus MHV-HP2 viruses for control (Scr) and treatment (ISG15) were compared after first log_10_-transforming the data to stabilize variances and to render the data normal, as required in the statistical analysis. Analyses of data at each hour, termed “by-hour analyses,” were performed using one-way ANOVA F-tests with Tukey HSD-adjusted *t*-tests used to adjust for multiple testing at each time point. Unless noted otherwise, differences were considered statistically significant here when *P* < 0.05, and all *P* values were two-sided. Weight-loss percent-of-baseline data were compared using Welch-adjusted one-way ANOVA tests by day for days 1–5 since non-constant variance was detected, and the three treatment means were subsequently compared using the Games-Howell (nonparametric) multiple comparison procedure. Weight-loss linear trends were assessed for MHV-HP2 and MHV-A59 treatments using mixed linear models with the heteroskedastic compound-symmetry covariance structure, which provided the optimal fit here. Cytokine data were compared using Welch-adjusted one-way ANOVA tests by hours post-infection since non-constant variance was detected. Unless noted otherwise, differences were considered statistically significant here when *P* < 0.05. All *P* values were two-sided. For all *in vivo* qPCR data, necroinflammatory foci, and serum cytokine analysis, GraphPad Prism Version 10.4.1 (532) for macOS (Boston, MA, USA) was used; data were analyzed using one-way ANOVA. *In vivo* viral loads were compared using the Mann-Whitney *U*-test (two-tailed) for each time point. Unless otherwise stated, significance thresholds are *P < 0.05, **P < 0.01, ***P < 0.001, ****P < 0.0001.

## Data Availability

All data supporting the findings of this study are included within the article and its supplemental material. The primary data are available from the corresponding author (sbaker1@luc.edu) upon reasonable request.

## SUPPLEMENTAL MATERIAL

Figure S1 jvi.00857-26-s0001.tiff MHV-HP2 forms smaller plaques than MHV-A59.

Figure S2 jvi.00857-26-s0002.tiff Accumulation of nsp3 and nsp4 in cells infected with MHV-A59 vs MHV-HP2.

Figure S3 jvi.00857-26-s0003.tiff Evaluating ISG15 expression and silencing in two distinct cell types.

Figure S4 jvi.00857-26-s0004.tiff MHV-HP2 has a transient replication delay that is not rescued by ISG15 silencing.

Figure S5 jvi.00857-26-s0005.tiff MHV-HP2 replication is attenuated compared to MHV-A59 in WT BMDMs and in BMDMs derived from mice harboring knockouts of type I IFN receptor and ISG15.

Figure S6 jvi.00857-26-s0006.tiff MHV-A59 induces greater levels of cytokine induction than MHV-HP2.

Supplemental legends jvi.00857-26-s0007.docx Legends for Fig. S1 to S6.

ASM does not own the copyrights to Supplemental Material that may be linked to, or accessed through, an article. The authors have granted ASM a non-exclusive, world-wide license to publish the Supplemental Material files. Please contact the corresponding author directly for reuse.
