# Supplementary material for: A chimeric murine coronavirus with altered deISGylase and proteolytic activities exhibits attenuated viral pathogenesis
Source: J Virol. 2026 Sep 30;100(10):e00857-26. doi: 10.1128/jvi.00857-26 (PMC13712883; doi:10.1128/jvi.00857-26)
Supplement: Supplementary file 1 [file jvi.00857-26-s0007.docx]

**Supplementary Figures**

**S1. MHV-HP2 forms smaller plaques than MHV-A59**

17Cl-1 cells were infected with either MHV-A59 or MHV-HP2 and overlaid with Noble agar. Plaque development was allowed to proceed for 2 dpi or 3 dpi. (A) Representative images of plaques at 2 and 3 dpi. (B) Quantification of plaque diameters (mm) at each time point. Each violin plot represents the distribution of individual plaque sizes; dashed lines indicate the median and quartiles. (****) p<0.0001. dpi, days post-infection.

**S2 (related to Figure 3C). Accumulation of nsp3 and nsp4 in cells infected with MHV-A59 vs MHV-HP2**

Densitometric analysis was performed on representative Western blots of nsp3 and nsp4. Band intensity was expressed as arbitrary units (AU).

**S3. Evaluating ISG15 expression and silencing in two distinct cell types.**

17Cl-1 cells and DBT cells were either mock-transfected or transfected with non-targeting siRNA or ISG15-targeting siRNAs. After 48 hours, cell lysates were harvested for Western blot analysis. Data are representative of n=2 biological replicates.

**S4 (related to Figure 4). MHV-HP2 has a transient replication delay that is not rescued by ISG15 silencing.**

DBT cells were transfected with either non-targeting or ISG15-targeting siRNAs (siRNA-Scr or siRNA-ISG15, respectively) 2 days prior to infection either with MHV-A59 or MHV-HP2 at an MOI of 0.1. At the indicated times, cell lysates were harvested for Western blot analysis (A) and cell-free supernatants were collected for plaque assay assessment (B). Data are representative of n=2 biological replicates, with PFU data depicting means (+/- SD) of technical triplicates. Plaque assay data were analyzed via one-way ANOVA on the log10-virus-titer data separately for each of the time points, using Tukey HSD adjusted F and t-tests to perform the depicted pairwise tests. (*) p<0.01; (**) p<0.01; (***) p<0.001; (****) p<0.0001; (ns) not significant. h.p.i, hours post infection.

**S5. MHV-HP2 replication is attenuated compared to MHV-A59 in WT BMDMs and in BMDMs derived from mice harboring knockouts of type I IFN receptor and ISG15**

(A, B) BMDMs generated from bone marrow isolated from WT C57BL/6 mice were infected with either MHV-A59 or MHV-HP2 at MOI of 1 (A) or MOI of 0.1 (B). Cell-free supernatants were collected at the indicated time points and subjected to plaque assay on 17Cl-1 cells. (C, D) Cell-free supernatants from (B) were assayed via CBA to measure levels of IFN-⍺ (C) and IFN-β (D). (E, F) BMDMs generated from bone marrow isolated from IFNAR-/- (E) and ISG15-/- (F) mice were infected with either MHV-A59 or MHV-HP2 at MOI of 1 and cell-free supernatants were collected at the indicated time points and subjected to plaque assay on 17Cl-1 cells. Plaque assay data are presented as the means (+/- SD) of technical triplicates and are representative of n=2 biological replicates. Data were analyzed via independent-sample t-tests by timepoint and confirmed using two-way ANOVA and a “within time analysis”. To account for concerns of multiple testing, α=0.01 was used as the cut-off to declare significance. Significance thresholds: (*) p<0.01; (**) p<0.001; (***) p<0.0001; (ns) not significant. CBA data are presented as the means (+/- SD) of n=2 biological replicates. Cytokine data were compared using Welch-adjusted one-way ANOVA tests by hours post infection. Significance thresholds: (*) p<0.05; (**) p<0.01; (***) p<0.001; (****) p<0.0001; (ns) not significant.

**S6 (related to Figure 6). MHV-A59 induces greater levels of cytokine induction compared to MHV-HP2.**

Sera from mice in Figure 6 were collected on the indicated day post-infection, heat-inactivated, and profiled for the expression of IL-1β (A), TNF-⍺ (B), IL-6 (C), and IFN-⍺ (D) via CBA. Data are represented as the mean (+/- SEM) of 5 mice, and statistical significance was calculated via ordinary one-way ANOVA. Significance thresholds: (*) p<0.05; (***) p<0.001; (ns) not significant.
